# Acute ER stress regulates amyloid precursor protein processing through ubiquitin-dependent degradation

**DOI:** 10.1038/srep08805

**Published:** 2015-03-05

**Authors:** Eun Sun Jung, HyunSeok Hong, Chaeyoung Kim, Inhee Mook-Jung

**Affiliations:** 1 Department of Biochemistry & Biomedical Science, Seoul National University College of Medicine, Seoul, 110-799, Republic of Korea; 2 Medifron DBT, Inc., Gyeongi, 425-838, Republic of Korea

## Abstract

Beta-amyloid (Aβ), a major pathological hallmark of Alzheimer's disease (AD), is derived from amyloid precursor protein (APP) through sequential cleavage by β-secretase and γ-secretase enzymes. APP is an integral membrane protein, and plays a key role in the pathogenesis of AD; however, the biological function of APP is still unclear. The present study shows that APP is rapidly degraded by the ubiquitin-proteasome system (UPS) in the CHO cell line in response to endoplasmic reticulum (ER) stress, such as calcium ionophore, A23187, induced calcium influx. Increased levels of intracellular calcium by A23187 induces polyubiquitination of APP, causing its degradation. A23187-induced reduction of APP is prevented by the proteasome inhibitor MG132. Furthermore, an increase in levels of the endoplasmic reticulum-associated degradation (ERAD) marker, E3 ubiquitin ligase HRD1, proteasome activity, and decreased levels of the deubiquitinating enzyme USP25 were observed during ER stress. In addition, we found that APP interacts with USP25. These findings suggest that acute ER stress induces degradation of full-length APP via the ubiquitin-proteasome proteolytic pathway.

Calcium plays an important role in regulating a great variety of brain processes. Degenerating neurons in brains of patients with Alzheimer's disease (AD) showed increased level of calcium^1^. Amyloid precursor protein (APP) is an integral membrane protein and plays a key role in the pathogenesis of AD. Even though several lines of evidence report that calcium dyshomeostasis affects APP processing^2^,^3^, the distinct importance of this process has not been elucidated in detail. APP is processed in the endoplasmic reticulum (ER) and transported through the secretory pathway to the plasma membrane where it is cleaved by α-secretase to produce the neuroprotective sAPPα^4^. Neurotoxic beta-amyloid (Aβ) is generated after sequential cleavage of APP by β-secretase (BACE1) and γ-secretase in the ER and Golgi/trans-Golgi-network (TGN)^5^,^6^. The ER is a subcellular organelle responsible for calcium homeostasis, protein folding, and transport^7^. Therefore, the study of subcellular localization of APP processing is important to elucidate AD pathogenesis.

The ubiquitin-proteasome system (UPS) is the major intracellular pathway for protein turnover control in eukaryotic cells^8^ and is closely linked to various neurodegenerative diseases such as AD, Huntington's disease (HD), Parkinson's disease (PD), prion diseases, as well as amyotrophic lateral sclerosis (ALS)^9^. Recent evidences suggest that protein misfolding and aggregation are common causes and pathological changes in diverse neurodegenerative disorders. AD is a progressive neurodegenerative disorder, histologically characterized by the accumulation of extracellular amyloid plaques and intraneuronal neurofibrillary tangles in the brain. Ubiquitin has been shown to accumulate in both plaques and tangles in the AD brain^10^,^11^,^12^. The UPS plays a pivotal role in the ER stress-induced degradation of misfolded proteins^13^. Increased levels of aberrant ER proteins rapidly activate both the unfolded protein response (UPR) and ER-associated degradation (ERAD)^14^,^15^. Abnormal calcium homeostasis is one of the factors that induce ER stress and dysfunction^16^. It is known that Aβ increases intracellular calcium levels and sustained stimulation of Aβ results in chronic ER stress in patients with AD^17^,^18^,^19^. Moreover, Aβ is implicated in the pathogenesis of AD through the impairment of proteasome function^20^,^21^. It has been reported that proteasome activity is altered in AD brains and dysregulation of proteasome system may be closely involved in AD pathogenesis^22^,^23^. Recent studies suggest that ER stress, along with abnormal calcium homeostasis, is central pathological events affecting APP processing in AD^24^,^25^. Therefore, it is not difficult to suppose that chronic ER stress and abnormal regulation of UPS contribute to the progression of AD. However, the relationship between APP processing and UPS pathway under acute ER stress is poorly understood. In this study, we show the dramatic degradation of APP through the UPS, under acute ER stress condition, by the elevation of intracellular calcium level.

## Results

### Effects of increased levels of intracellular calcium on APP processing

**Figure 1:**
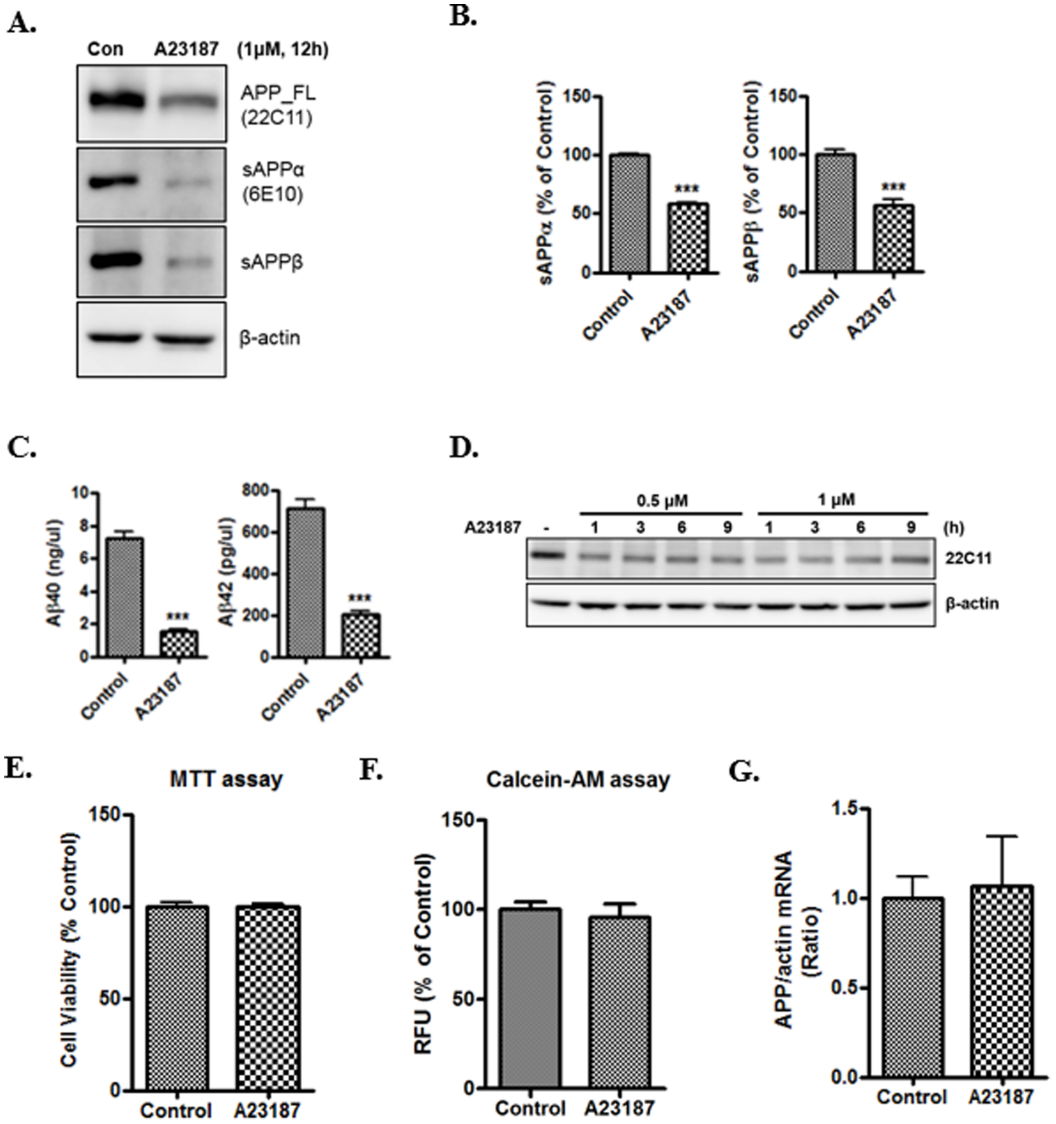
Effects of increased intracellular calcium levels on APP processing. 7w-PSML cells were treated with A23187 (1 μM) for 12 h. (A), Cellular lysates are blotted for full-length APP (22C11 antibody). Loading control; β-actin. Conditioned media were blotted for sAPPα (6E10 antibody) and sAPPβ. (B), Quantification of sAPPα (6E10 antibody) and sAPPβ. (C), Aβ40 and Aβ42 measured in conditioned media. (D), 7w-PSML cells were incubated for various times with A23187 (0.5 μM or 1 μM). (E, F). Treatment with A23187 did not affect cell viability. Cell viability was measured by the MTT assay and calcein-AM assay. (G), Total APP mRNA level is unchanged in HEK cells treated with A23187 for 12 h. Statistical significance was tested by unpaired t-test (n = 4, ***P < 0.001 versus control group). All the gels were run under the same experimental conditions. Full-length images are presented in the supplementary information.

Calcium ionophore A23187 is widely used to increase intracellular calcium levels. To examine whether calcium stress affects APP processing, 7w-PSML cells were treated with A23187 (1 μM) for 12 h. 7w-PSML is a CHO cell line stably transfected with both wild-type APP and mutant presenilin-1 (M146L)^26^, which is an efficient model for detection of APP and its metabolites, containing Aβ40 and Aβ42. We confirmed that A23187 increased intracellular calcium levels in 7w-PSML cells (Supplementary figure 1). A23187-treated 7w-PSML cells showed more than 50% reduction in APP, sAPPα, and sAPPβ levels at the 12 h treatment time (Figure 1 A and B). Aβ40 and Aβ42 levels were also significantly reduced after A23187 treatment (Figure 1C). In addition, we detected a dramatic reduction in APP with a low concentration (0.5 μM) of A23187 at a short exposure time (1 h) by western blot analysis (Figure 1D). When we tested the cytotoxic effect of A23187 using the MTT assay and calcein-AM assay, A23187 did not affect cell viability and had no cytotoxicity in our experimental conditions (Figure 1E, F). To determine whether APP is down-regulated at the transcriptional level by A23187 treatment, real-time RT-PCR for APP mRNA was performed. We observed no significant difference in the APP transcript levels in A23187-treated cells compared with control cells (Figure 1G). Taken together, A23187-induced calcium influx down-regulated APP protein, but not mRNA levels in 7w-PSML cells, indicating post-transcriptional regulation of APP by increased intracellular calcium levels.

### Acute ER stress induces APP degradation through ubiquitin-proteasome pathway

**Figure 2:**
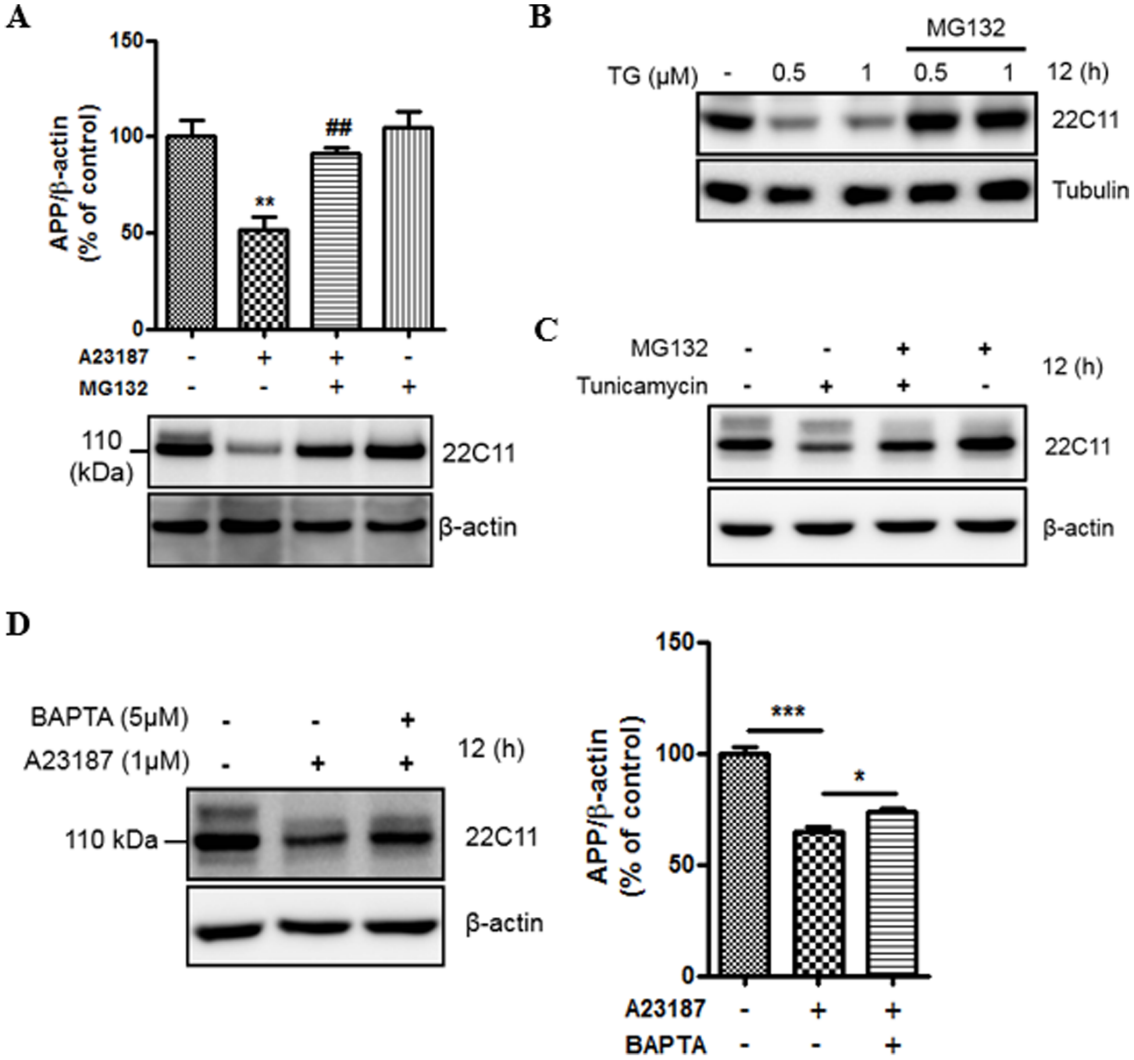
ER stress induces APP degradation. (A), 7w-PSML cells pretreated with MG132 or vehicle for 30 min were exposed to A23187 for 12 h. Cells were harvested for immunoblotting with anti-22C11 (APP) and anti-β-actin. The bottom panel shows a representative western blot, and the top panel shows the quantification based on densitometry. Statistical significance was analyzed by one-way ANOVA followed by a Tukey's multiple-comparison test. (n = 4, **P < 0.01 versus control group; ^##^P < 0.01 versus A23187-treated group) (B, C), Pretreatment with MG132, a proteasome inhibitor, protects the APP protein from ER stress-mediated degradation. 7w-PSML cells were pre-treated with MG132 for 30 min followed by treatment with thapsigargin (B) or tunicamycin (C) for 12 h, and harvested for western blot analysis to analyze expression levels of APP (22C11). (D), 7w-PSML cells were pretreated for 60 min with BAPTA/AM (5 μM) before a 12 h treatment with A23187 (1 μM). Chelation of the intracellular calcium with BAPTA prevented degradation of APP. Statistical significance was analyzed by one-way ANOVA followed by a Tukey's multiple-comparison test (n = 5, ***P < 0.001 and *P < 0.05). All the gels were run under the same experimental conditions. For each experiment, APP level was quantified by densitometry and normalized to β-actin loading control. Full-length images are presented in the supplementary information.

Because the decrease in APP protein levels did not correlate with a corresponding decrease in the APP mRNA level after A23187 treatment, we examined the underlying mechanism of A23187-induced APP protein reduction. To investigate if the A23187-induced decrease in APP levels could result from a proteasome-dependent APP degradation, we pretreated 7w-PSML cells with MG132 (10 μM), a proteasome inhibitor, 30 min before A23187 treatment. As shown in the representative western blot (Figure 2A), A23187-induced reduction in APP levels was dramatically restored by pretreatment with MG132 (Figure 2A). This result was also confirmed in a mouse hippocampal neuronal cell line, HT22, treated under the same conditions (Supplementary figure 2). In addition, we pretreated 7w-PSML cells with lactacystin (10 μM), a more specific inhibitor of the 26S proteasome than MG132^27^, 30 min before A23187 treatment. Pretreatment with lactacystin prevented the reduction in levels of APP protein by A23187 treatment (data not shown). Furthermore, we investigated the level of nicastrin, which is known to be regulated by proteasomes^28^,^29^ and processed in ER transport to the plasma membrane like APP, during ER stress. Nicastrin is an essential component of the γ-secretase complex^30^. We observed that MG132 also rescued the A23187-induced degradation of nicastrin (Supplementary figure 3). These data suggest that A23187-induced proteasomal degradation may be a general effect on most plasma membrane proteins and not an APP-specific effect. Next, we examined the effect of other ER stress inducers including thapsigargin (an inhibitor of the ER calcium pump; SERCA) and tunicamycin (an inhibitor of protein glycosylation). We observed that the inhibition of proteasomes restored the reduced levels of APP protein caused by both thapsigargin and tunicamycin (Figure 2B, C). Since increase in intracellular calcium levels by both ionophore and ER-stress inducers affect APP protein levels, the calcium chelator BAPTA/AM was used to confirm the correlation between intracellular calcium and APP protein levels. Pretreatment with BAPTA/AM prevented the effect of the ionophore on APP protein (Figure 2D). These results suggest that ER-stress related altered calcium homeostasis induces APP degradation via the proteasome.

### APP is preferentially degraded through the proteasome pathway in response to acute ER stress

**Figure 3:**
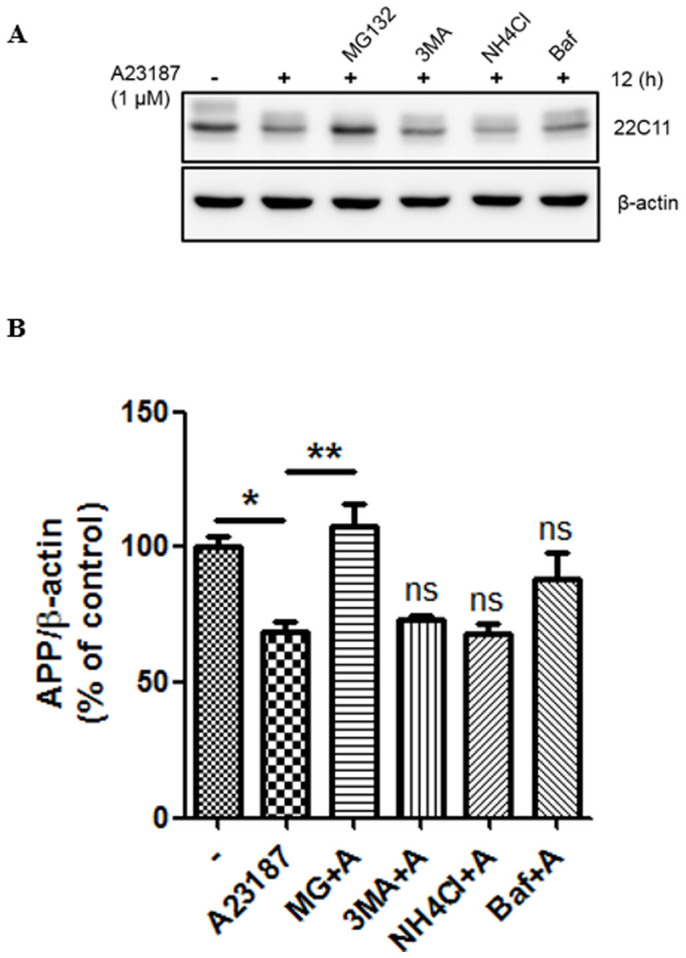
APP is preferentially degraded through the proteasome pathway in response to ER-stress. (A), CHO cells were pretreated with 10 μM MG132, 1 mM 3MA, 20 mM NH_4_Cl, or 10 nM Bafilomycin (Baf) for 30 min and later treated with A23187 (1 μM) for 12 h. Representative western blots show APP (22C11) and β-actin expression. (B), Quantification of APP. Statistical significance was tested by one-way ANOVA followed by a Tukey's multiple comparison test (n = 5; *P < 0.05, **P < 0.01). All the gels were run under the same experimental conditions. For each experiment, APP level was quantified by densitometry and normalized to β-actin loading control. Full-length images are presented in the supplementary information.

In mammalian cells, autophagy and the ubiquitin-proteasome system (UPS) are the major pathways of intracellular protein degradation^31^. To confirm whether APP is degraded through the ubiquitin-proteasome system under ER-stress conditions, CHO cells were pretreated with various inhibitors of the proteolytic pathway namely, 3-methyladenine (3MA), ammonium chloride (NH_4_Cl), and bafilomycin A1 for 30 min and then treated with calcium ionophore A23187 for 12 h. 3MA is an autophagy inhibitor, and NH_4_Cl and bafilomycin A1 are inhibitors of lysosomal function. Because calcium ionophore is known to induce autophagy^32^,^33^, we checked the levels of the autophagy marker protein, LC3-II (Supplementary figure 4). A23187 induced accumulation of LC3-II; this accumulation was suppressed by the autophagy inhibitor, 3MA. A23187-induced accumulation of LC3-II was increased by both NH_4_Cl and bafilomycin A1, which are autolysosome inhibitors^34^,^35^ (Figure S4A). Moreover, A23187 also significantly increased the ratio of LC3-II/LC3-I, which was decreased by 3MA and increased by both NH_4_Cl and bafilomycin A1 (Figure S4B). Interestingly, calcium ionophore mediated degradation of APP was prevented only by the proteasome inhibitor, MG132 (Figure 3). Therefore, this data suggests that APP is preferentially degraded by proteasomes in response to acute ER stress.

### Polyubiquitination of APP by intracellular calcium overload

**Figure 4:**
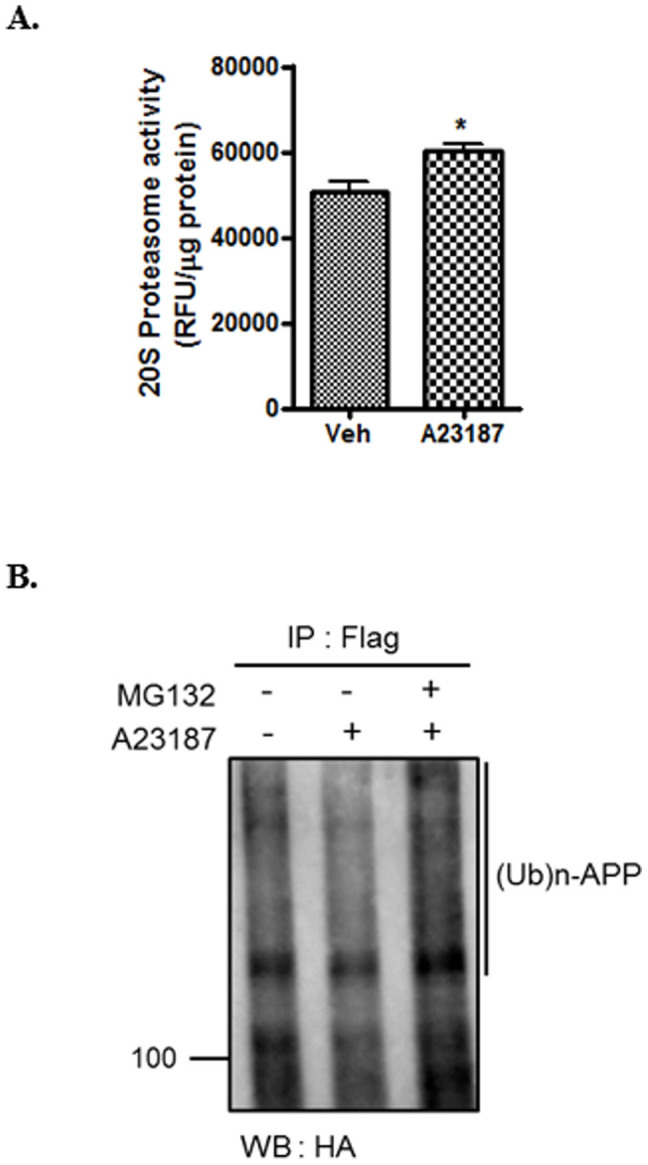
Polyubiquitination of APP by intracellular calcium overload. (A), CHO cells were treated with A23187 (1 μM) for 12 h. Proteasome activity measured using a 20S proteasome activity kit (APT280; Millipore). (B), CHO cells were co-transfected with HA-tagged ubiquitin (Ub-HA) and Flag-tagged APP (Flag-APP). After 24 h, CHO cells were pre-incubated with MG132 or vehicle for 30 min followed by incubation with A23187 for 12 h, and equivalent lysates were immunoprecipitated for Flag (APP) and blotted with antibodies to HA for HA-ubiquitin. Statistical significance was tested by one-way ANOVA followed by unpaired t-test (n = 4. *P < 0.05 versus vehicle). Full-length images are presented in the supplementary information.

Since proteasome pathway is involved in APP degradation under ER stress, proteasome activities after calcium ionophore treatment were measured. Calcium ionophore treatment significantly increased the activities of the 20S proteasome by nearly 20% (Figure 4A). Because poly-ubiquitination is an important process for targeting proteins to the proteasome, we investigated whether APP degradation undergoes this process. CHO cells were co-transfected with Flag-tagged APP and HA-tagged ubiquitin, and pretreated with MG132 or vehicle for 30 min before ionophore treatment for 12 h, followed by immunoprecipitation with anti-Flag antibodies for Flag-tagged APP. Immunoblotting was then performed with antibodies against HA for HA-tagged ubiquitin. We observed poly-ubiquitination of APP in MG132 treated cells (Figure 4B). These data indicate that the pretreatment of CHO cells with MG132 inhibited the calcium ionophore induced degradation of ubiquitinated APP.

### ER stress-mediated APP degradation is associated with ERAD pathway

**Figure 5:**
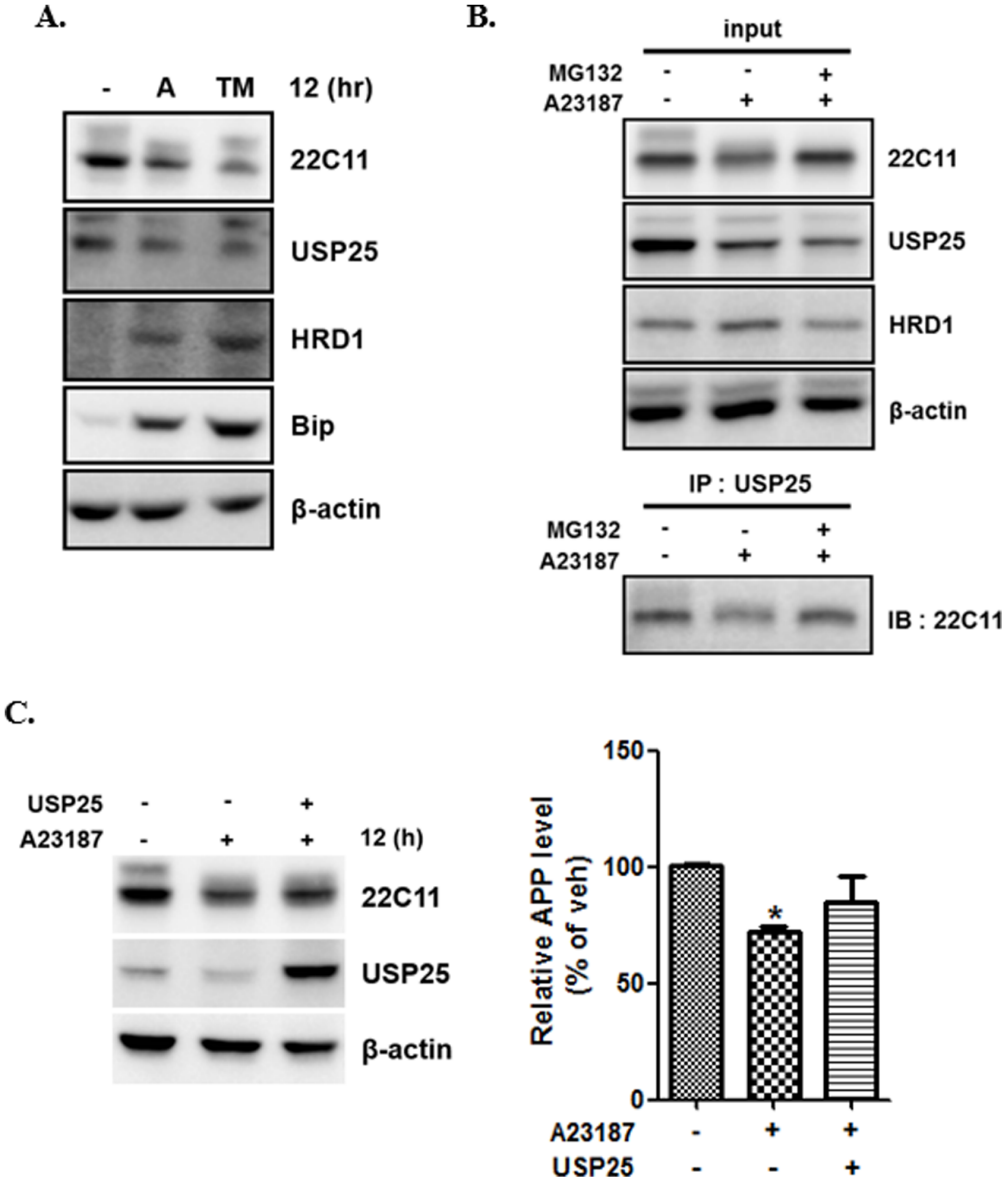
ER stress regulates APP stability via ER-associated protein degradation pathway. (A), CHO cells were treated with A23187 or tunicamycin for 12 h. Cells were then harvested for immunoblotting with anti-22C11 (APP), anti-USP25, anti-HRD1, anti-Bip, and anti-β-actin antibodies. (B), CHO cells pre-incubated with MG132 or vehicle for 30 min were exposed to A23187 for 12 h. Whole-cell extract of CHO cells was subjected to immunoprecipitation (IP) with anti-USP25 antibody and then immunoblotted with anti-22C11 (APP), anti-USP25, anti-HRD1, and anti-β-actin antibodies. (C), CHO cells were treated with A23187 for 12 h after transfection with USP25. The total cell lysates were analyzed by western blotting with the antibodies. For each experiment, APP level was quantified by densitometry and normalized to β-actin loading control. Statistical significance was analyzed by one-way ANOVA followed by a Tukey's multiple-comparison test. (n = 3. *P < 0.05 versus vehicle). All the gels were run under the same experimental conditions. Full-length images are presented in the supplementary information.

The unfolded protein response (UPR) is the major compensatory and defense mechanism against ER stress^36^. UPR is known to enhance degradation of misfolded proteins through the up-regulation of molecules involved in the ER-associated degradation (ERAD) pathway. We sought to determine whether ER stress affected the level of ERAD-related proteins. Levels of APP and the deubiquitin enzyme, ubiquitin-specific protease 25 (USP25) were decreased; however, levels of human homolog of yeast Hrd1p/Der3p (HRD1), an ERAD-associated E3 ubiquitin ligase, and binding immunoglobulin protein (BiP) increased in CHO cells with ionophore or tunicamycin treatment for 12 h (Figure 5A).

To elucidate the interaction between USP25 and APP, we performed immunoprecipitation using anti-USP25 antibodies and observed that APP interacted with USP25. After treatment with the ionophore, USP25 interaction with APP was decreased (Figure 5B). We investigated whether USP25 prevents APP degradation under ER-stress conditions. We confirmed that A23187-induced APP degradation was rescued by the overexpression of USP25 (Figure 5C). Together, these findings suggest that the activation of A23187-mediated ERAD resulted in APP degradation, and that USP25 inhibits APP degradation by the proteasome.

### Inhibition of the proteasomal pathway causes an accumulation of APP in the ER under ER-stress

**Figure 6:**
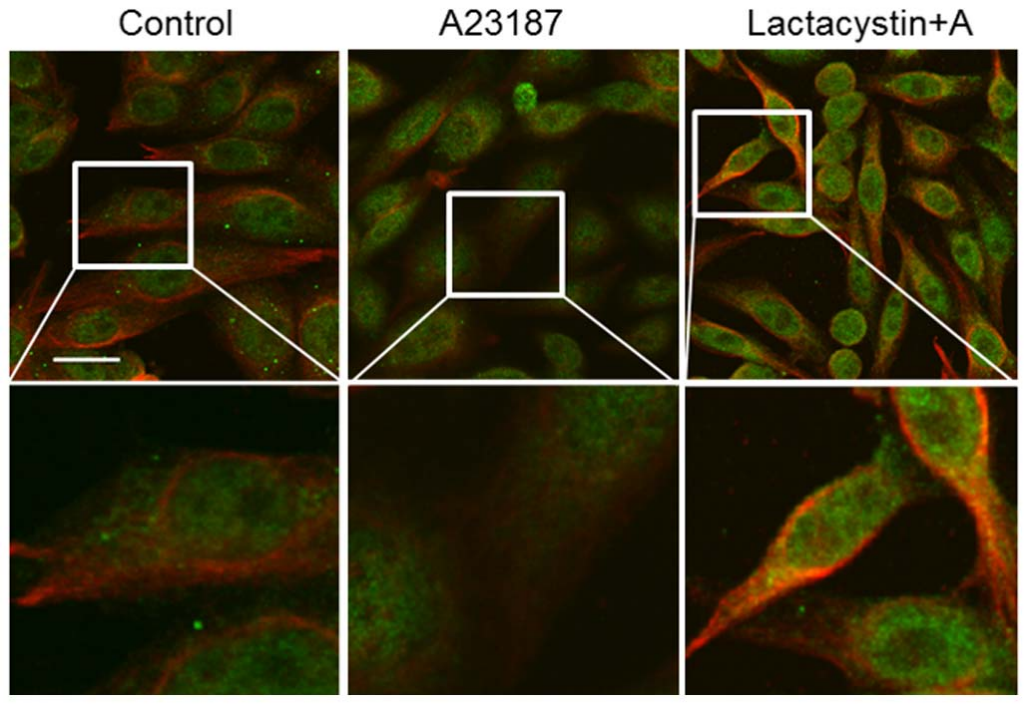
Proteasome inhibition induces the accumulation of APP in the ER under ER-stress conditions. CHO cells pre-treated with proteasome inhibitor, lactacystin, or vehicle for 30 min were exposed to A23187 for 12 h. Cells were fixed and stained with antibodies against APP (22C11) and calnexin, followed by secondary antibodies conjugated with Alexa 594 and Alexa 488, respectively. Scale bar; 20 μm.

Because APP is produced in the ER and transits to the Golgi, we examined whether APP is accumulated in the ER under ER stress by using immunofluorescence staining. A23187 treated cells showed that the co-localization of APP and the ER marker calnexin increased in the presence of the proteasome inhibitor, lactacystin (Figure 6). These data suggest that the inhibition of ER stress-mediated APP degradation by the proteasome inhibitor lactacystin may lead to the accumulation of APP in the ER.

## Discussion

It is known that the mature APP promotes Aβ generation^37^, which occurs in the ER or TGN^6^,^38^. Since irregular processing of APP results in the overproduction of Aβ, it is important to elucidate the mechanism that regulates APP processing. Several lines of evidence suggest that calcium dysregulation influences Aβ generation and AD pathogenesis^39^,^40^,^41^. Because the ER is a pivotal cellular organelle in regulating calcium homeostasis^16^, and disruption of calcium homeostasis is generally related with ER stress^42^, the ER stress-induced changes in APP processing were examined in this study. We have described here that the ER stress-induced degradation of APP is mediated by the ubiquitin-proteasome system. Calcium ionophore dramatically reduced full-length APP, sAPPα, sAPPβ, and Aβ levels (Figure 1). Calcium ionophore-mediated reduction in APP was completely inhibited by proteasome inhibitors, MG132 (Figure 2A) or lactacystin (data not shown), but not by autophagy inhibitors, 3MA or bafilomycin A1 (Figure 3). These results suggest a specific role of the UPS against degradation of APP by ER stress. Unlike the proteasomal degradation, autophagy is mainly involved in eliminating long-lived proteins and other cytoplasmic contents^43^. Moreover, autophagy is a bulk degradation mechanism and usually induced during nutrient starvation^44^. On the other hand, UPS is a specific degradation system for most short-lived soluble proteins^8^. We showed that APP was rapidly degraded at earlier time point (1 h) after treatment with calcium ionophore A23187 (Figure 1D). A23187 is widely used to increase intracellular calcium levels, and several studies have reported that A23187 induces apoptosis^45^,^46^. We examined whether A23187 treatment affects cell death in our experimental system. The total protein visualized by Coomassie blue staining remained unchanged after treatment with A23187 (data not shown) and did not affect cell death (Figure 1E, F). Therefore, these data suggest that A23187-mediated reduction of APP levels is via UPS, and not because of cell death, under our experimental conditions. There are other inducers of ER-stress such as tunicamycin and thapsigargin^47^. Similar to A23187, we found that both tunicamycin and thapsigargin reduced the APP level, which was rescued by the proteasome inhibitor, MG132 (Figure 2B, C), suggesting that a reduction in APP is closely associated with disturbance of calcium homeostasis in the cells. Based on our data in figure 2, 3 and 5, ER stress affects the immature form of unglycosylated or/and unfolded/misfolded APP in the ER, which is a lower band of APP by detection with 22C11 antibody. Most of control cells without ER stress showed doublet band of APP, which means it contains both mature and immature forms of APP. Therefore, we focused on the immature form of APP processing to examine the relationship between ER stress and UPS degradation pathway on APP processing.

We observed that ERAD-associated proteins were regulated by the presence of the ER stress agents A23187 and tunicamycin. Recently, several studies have reported that APP is a substrate for both ERAD-associated ubiquitin E3 ligase HRD1 and ubiquitin-specific protease 25 (USP25)^48^,^49^. USP25 was decreased in CHO cells treated with ER stress inducers, whereas HRD1 and BiP were increased. BiP (GRP78) is an ER stress sensor, and functions in the retrograde transport of abnormal proteins across the ER membrane, destined for degradation by the proteasome^50^. The dramatic degradation of APP could be mediated by the synergistic effects of both decreased UPS25 and increased HRD1 levels under ER stress conditions. Acute, mild ER stress generally induces the expression of ER-resident chaperones and ERAD components for the adaptation and survival of cells, whereas sustained, excessive ER stress results in apoptosis or cellular dysfunction^51^. ER stress-related disturbance of calcium homeostasis can interfere with protein folding and subsequently leads to accumulation of misfolded or unfolded proteins in the ER, which activates the unfolded protein response (UPR) and prevents the cellular build-up of the toxic misfolded proteins^52^. AD is characterized by the accumulation and aggregation of misfolded proteins. APP, a protein involved in the progression of AD, is vulnerable to misfolding in the ER, and misfolded APP is targeted to the ERAD^53^. In the present study, we observed the acute ER stress-mediated activation of proteasome (Figure 4A), and activated proteasome may be involved in the degradation of misfolded APP through ERAD. Proteasome normally protects cells from acute ER stress conditions, but impaired proteasome function has been reported in AD^22^. When CHO cells were treated with the proteasome inhibitor MG132, APP was co-localized with the ER marker calnexin after treatment with A23187 (Figure 6), indicating accumulation of APP in the ER under conditions of proteasomal dysfunction. In the ER compartment, BACE1 is initially synthesized, and partial BACE1-cleavage can still occur^54^. Moreover, γ-secretase is predominantly located in ER^55^. Several reports suggest that the intracellular Aβ is closely associated with ER stress, which is an early event in AD pathogenesis^56^,^57^. Furthermore, there is evidence that ER stress inhibits proteasome activity^58^,^59^. We, therefore, speculate that the abnormal accumulation of APP in the ER by proteasome dysfunction can increase to generate intracellular Aβ in pathological conditions of AD such as chronic ER stress.

Our results imply that acute ER stress induces expression of ERAD-associated chaperones, which rapidly degrade APP through the ubiquitin-dependent proteasomal pathway for elimination of unfolded or misfolded APP, and consequently prevents Aβ production. On the other hand, proteasome dysfunction by chronic ER stress causes APP accumulation in the ER and more Aβ generation. Our findings provide insight on the importance of APP processing under acute stress condition and the proteasome as a potential therapeutic target for treating AD.

## Methods

### Reagents and antibodies

A23187, tunicamycin, thapsigargin, MG132, lactacystin, bafilomycin, NH_4_Cl, 3-methyladenine (3MA), and BAPTA were purchased from Sigma-Aldrich (St. Louis, MO, USA). The following antibodies were used for immunodetection: anti-22C11, Nicastrin (Millipore, Billerica, MA, USA), 6E10 (Signet, Dedham, MA, USA), sAPPβ (Covance, Princeton, NJ, USA), anti-Flag (Sigma), HA (Cell Signaling Technology, Seoul, Korea), USP25 (Santa Cruz, Dallas, TX, USA), HRD1 (Abgent, Seoul, Korea), Calnexin (Enzo, Farmingdale, NY, USA), LC3B (Novus, Littleton, CO, USA), GAPDH (Abcam, Seoul, Korea), β-actin and tubulin (Sigma-Aldrich).

### Cell Culture and transfection

Chinese Hamster Ovary (CHO) cells, wild type human APP and mutant presenilin-1 (M146L) overexpressing CHO cells (7W-PSML, gifted from Dr. David Kang, at Florida State University), and human embryonic kidney 293 cells (HEK293) were cultured in Dulbecco's modified Eagle medium (DMEM; HyClone), supplemented with 10% fetal bovine serum (FBS; HyClone) and 0.1 mg/mL penicillin and streptomycin (P/S; Sigma-Aldrich) in a humidified incubator at 37°C and 5% CO_2_. CHO cells were transiently transfected with 1 μg/well (6 well-plates) plasmid DNAs using Lipofectamine™ LTX (Invitrogen, Carlsbad, CA, USA) according to the manufacturer's instruction.

### Visualization of intracellular calcium

The intracellular calcium was measured using the Fluo-4 Direct™ Calcium Assay Kit (Life technologies, USA) as per manufacturer's protocol. Briefly, the cells were plated in 96-well plates. Culture media was removed from plates and the cells were incubated with 5 mM Fluo-4 calcium reagent at 37°C for 1 h. The cells were treated with A23187, and intracellular calcium was observed using a fluorescence microscope (Olympus DP50) with a fluorescein isothiocyanate (FITC) filter set.

### MTT assay

The degree of cell viability was measured using the MTT assay. Twelve hours after the addition of the A23187, the cell culture media was replaced with 50 μL per well phenol red-free medium containing 2.5 μg/mL MTT for 2 h at 37°C, followed by the aspiration of the MTT solution. Cells were then solubilized by adding 140 μL of isopropanol per well for 30 min at 37°C. After incubation, plates were equilibrated to room temperature (RT) for an additional 30 min. The relative reduction of MTT was quantified by measuring optical density at 540 nm using a plate reader (Bio-Tek instruments, CA, USA).

### Calcein-AM assay

The degree of cytotoxicity was measured using the calcein-AM assay as previously described^60^. Briefly, the cells were plated in 96-well plates and treated with A23187 for 12 h. The cell culture media was removed from plates and the cells were incubated with 50 μL per well of 1 μM calcein-AM (Molecular Probes) in Opti-MEM solution at 37°C for 1 h. After incubation, the plate was washed and fluorescence was measured at 485 nm (excitation)/530 nm (emission) wavelengths.

### Western Blot

The cells were homogenized in RIPA buffer containing protease inhibitor cocktail (Sigma Aldrich), incubated on ice for 15 min, and sonicated for 5 sec. After centrifugation at 13,000 rpm for 10 min, the supernatant was collected, and protein concentration was determined by the BCA kit (Pierce, Rockford, IL, USA). Equal amounts of protein samples were separated on 6–10% SDS-PAGE or NuPAGE 4–12% Bis-Tris gel (Novex Life technologies, Seoul, Korea). Immunoreactivity was determined by enhanced chemiluminescence (ECL) (Amersham Pharmacia Biotech, Buckinghamshire, UK). The chemiluminescence signal was quantified with a digital image analyzer (LAS-3000, Fuji, Japan). The protein levels were normalized with β-actin.

### Immunoprecipitation

CHO cells were washed twice with phosphate-buffered saline (PBS), lysed with 1X hypotonic buffer (10 mM Tris-HCl; pH 7.4, 1 mM EDTA, 1 mM EGTA, 1 mM PMSF, protease inhibitor cocktail) containing 1% CHAPS. The cell lysates were then centrifuged at 13,000 rpm for 15 min at 4°C to discard cell debris. Supernatants were incubated with anti-Flag antibody (Sigma) overnight at 4°C. Dynabeads protein G (Life Technologies) were then added to the samples, and incubation was continued for 60 min at 4°C. Immunoprecipitates were washed four times with lysis buffer, and SDS sample buffer was added to the samples, which were subsequently heated and subjected to SDS-PAGE. Immuno-blotting was performed as described above.

### Aβ ELISA

Human Aβ40 and Aβ42 levels were quantified using a commercially available ELISA kit (IBL, Hamburg, Germany) according to the manufacturer's protocol. In brief, conditioned media from A23187-treated 7W-PSML cells were loaded onto plates coated with Aβ N-terminal-specific antibody and incubated with Aβ C-terminal-specific antibody overnight at 4°C. Aβ was detected by incubating for 1 h at RT with horseradish peroxidase-conjugated secondary antibody. ELISA plates were developed using a color reaction, and the absorbance was read at 450 nm using a plate reader (Bio-Tek instruments, CA, USA).

### 20S proteasome activity assay

Proteasome activity was measured using a 20S proteasome activity assay kit (APT280, Millipore) according to the instructions of manufacturer. Briefly, the assay was based on detection of the fluorophore 7-amino-4-methylcoumarin (AMC) after cleavage from the labeled substrate LLVY-AMC by the proteasome machinery. Protein samples (20 μM) were incubated for 60 min at 37°C with proteasome substrate, LLVY-AMC. The free AMC fluorescence was quantified at 360/460 nm in a fluorimeter.

### RNA Isolation and Real-time PCR

Total RNA was isolated from HEK293 cells using the Qiagen RNeasy kit (Qiagen, Valencia, CA, USA). Equal amounts of total RNA (2 μg) from each sample were converted into cDNA using Maxime RT Premix_Oligo dT primer kit (Intron Biotechnology, Seoul, Korea). Real-time PCR for the expression level of APP mRNA was performed using an ABI stepone 2.1 (Applied Biosystems, Foster City, CA, USA). Specific primers for APP (forward: GCAGTGAGAAGAGTACCAAC/reverse: ACCTCATCACCATCCTCATC) and actin (forward: AGCCTCGCCTTTGCCGA/reverse: CTGGTGCCTGGGGCG) were used. Actin gene was used as an endogenous control to standardize the amount of RNA.

### Immunocytochemistry

After two washing steps with prewarmed PBS, cells were fixed in 4% paraformaldehyde in PBS for 10 min at RT, permeabilized with 0.1% Triton X-100, blocked with PBS containing 5% bovine serum albumin (BSA), and incubated with primary antibodies overnight at 4°C. This was followed by incubation with Alexa Fluor conjugated secondary antibodies for 1 h at RT. Fluorescent signals were visualized by laser scanning confocal microscopy (LSCM; Olympus Fluoview 300).

### Data analysis and statistics

Data are presented as mean ± SEM. Differences between the groups were evaluated by t-test or one-way ANOVA with Tukey's multiple comparison tests using the GraphPad Prism 5 software (CA, USA). Significance was accepted at P < 0.05.

## Supplementary Material

Supplementary Information Supplementary Information
