# Supplementary material for: Acute ER stress regulates amyloid precursor protein processing through ubiquitin-dependent degradation
Source: Sci Rep. 2015 Mar 5;5:8805. doi: 10.1038/srep08805 (PMC5390087; doi:10.1038/srep08805)

Supplementary Information

Acute ER stress regulates amyloid precursor protein processing through ubiquitin-dependent degradation

Eun Sun Jung[1], HyunSeok Hong[2], Chaeyoung Kim[1], Inhee Mook-Jung[1]*

[1] Department of Biochemistry and Biomedical Sciences, Seoul National University, College of Medicine, 103 Daehak-ro, Jongro-gu, Seoul 110-799, Korea

[2] Medifron DBT, Inc., 1277 Singil-dong, Danwon-gu, Ansan si, Gyeongi-do, 425-838, Korea

*Corresponding Author:

Inhee Mook-Jung, PhD
Department of Biochemistry and Biomedical Sciences, Seoul National University, College of Medicine, 103 Daehak-ro, Jongro-gu, Seoul 110-799, Korea
E-mail) inhee@snu.ac.kr
Phone number) +82-2-740-8245
Fax number) +82-2-3672-7352

Supplement Figure 1.

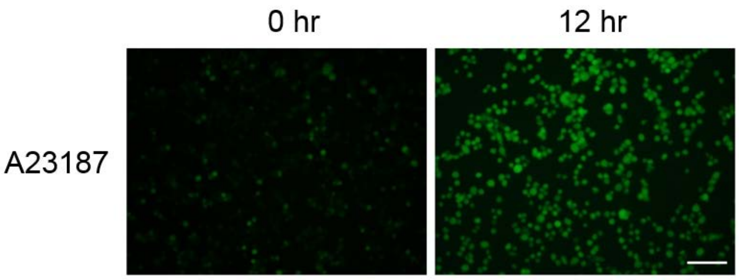

Fig S1. A23187 increases intracellular calcium levels in 7w-PSML cells. Fluo-4-loaded 7w-PSML cells were incubated with 1 µM A23187 (calcium ionophore). The staining images were obtained on a fluorescent microscope at the indicated times. Scale bar, 100 µm.

Supplement Figure 2.

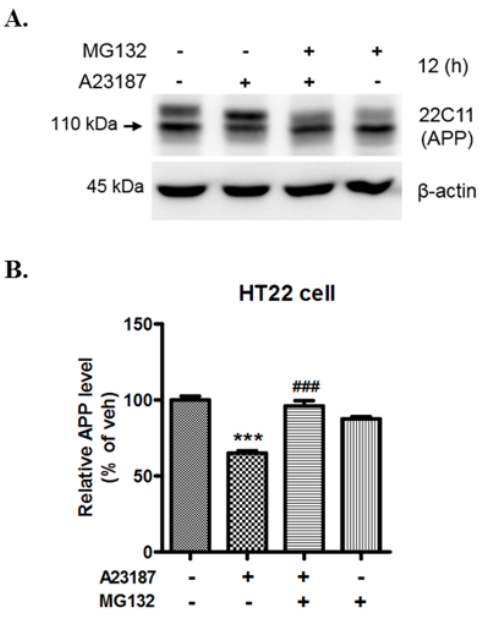

Fig S2. ER stress induces APP degradation in HT22 cell lines. A. HT22 cells, mouse hippocampal neuronal cell line, pretreated with MG132 or vehicle for 30 min were exposed to A23187 for 12 h. Cells were harvested for immunoblotting with anti-22C11 (APP) and anti-β-actin. B. The quantification of APP based on densitometry. Statistical significance was analyzed by one-way ANOVA followed by a Tukey's multiple-comparison test. (n=4, ***P < 0.001 versus control group; ###P < 0.001 versus A23187-treated group). For each experiment, APP level was quantified by densitometry and normalized to β-actin loading control. Full-length images are presented in supplementary information.

Supplement Figure 3.

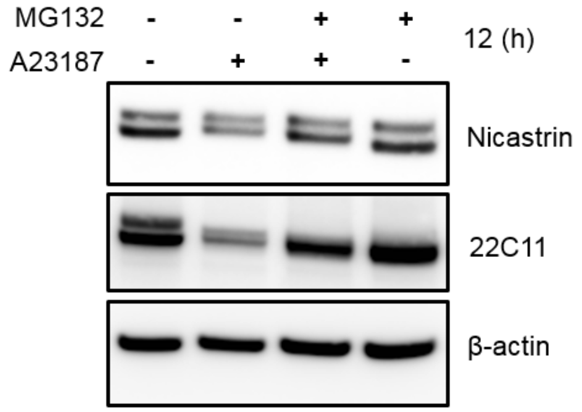

Fig S3. Pretreatment with MG132, a proteasome inhibitor, protects the Nicastrin protein from ER stress-mediated degradation. 7w-SPML cells pretreated with MG132 or vehicle for 30 min were exposed to A23187 for 12 h. Cells were harvested for immunoblotting with anti-22C11 (APP), anti-nicastrin and anti-β-actin. Full-length images are presented in supplementary information.

Supplement Figure 4 Related to Fig 3.

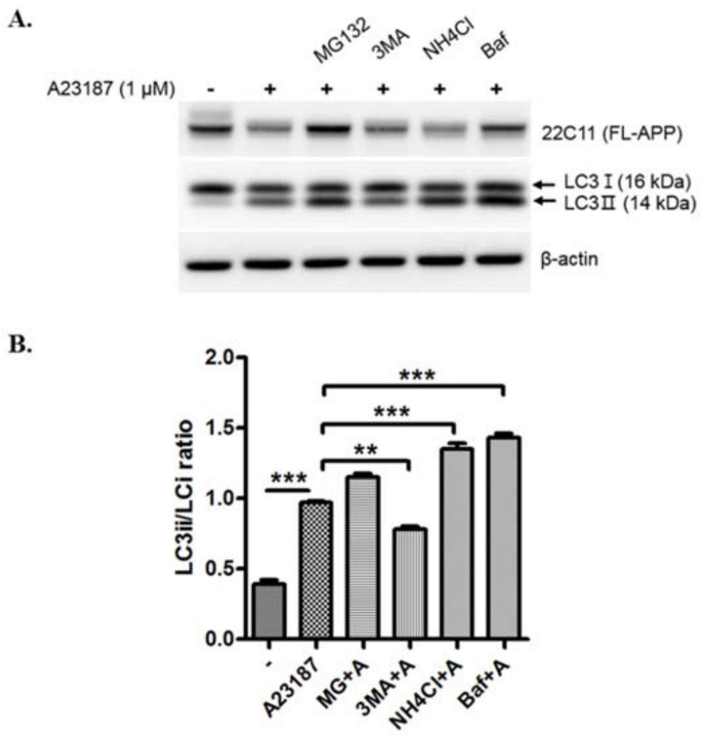

Fig S4. A, CHO cells were pretreated with 10 μM MG132, 1 mM 3MA, 20 mM NH4Cl or 10 nM Bafilomycin (Baf) for 30 min and then treated with A23187 (1 μM) for 12 h. Representative

Western blots were detected with antibodies targeting APP (22C11), LC3 and β-actin. B, Quantification of LC3ii/LC3i ratio. Statistical significance was tested by one-way ANOVA followed by a Tukey's multiplecomparison test (n=3; $^{**}P < 0.01$, $^{***}P < 0.001$). All the gels were run under the same experimental conditions. Full-length images are presented in supplementary information.

Full-length Western Blot image

Figure 1A.

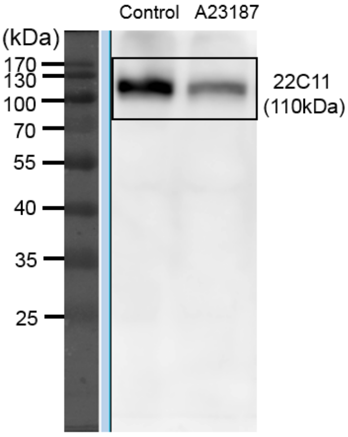

10% Glycine gel

Figure 1A.

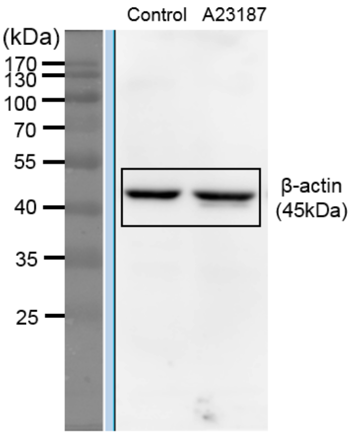

Figure 1A.

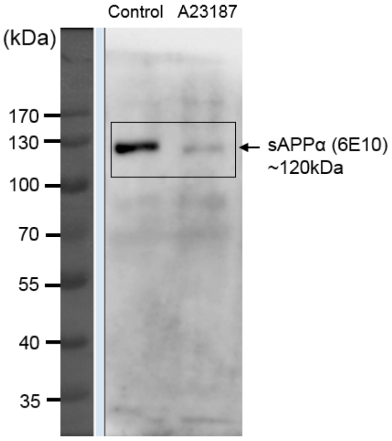

8% Glycine gel

Figure 1A.

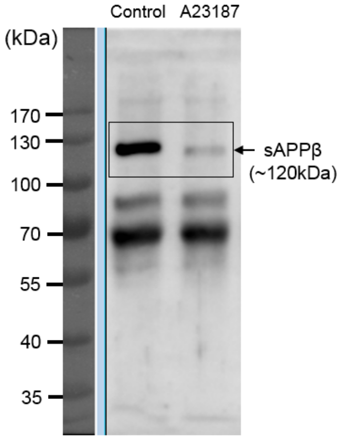

Figure 1D.

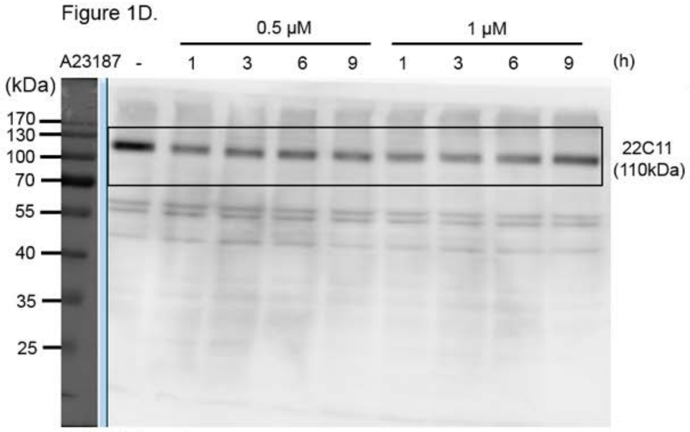

10% Glycine gel

Figure 1D.

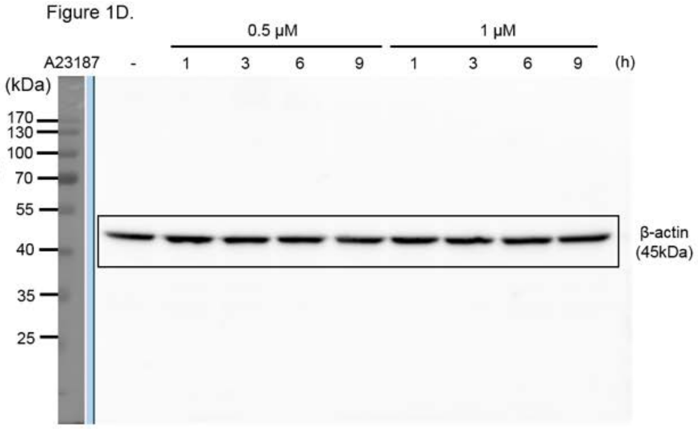

Figure 2A.

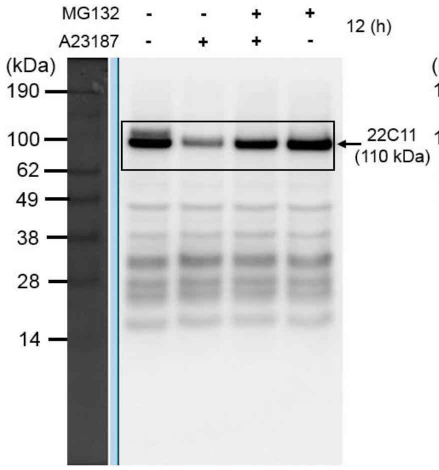

4-12% NuPAGE (Novex)

Figure 2A.

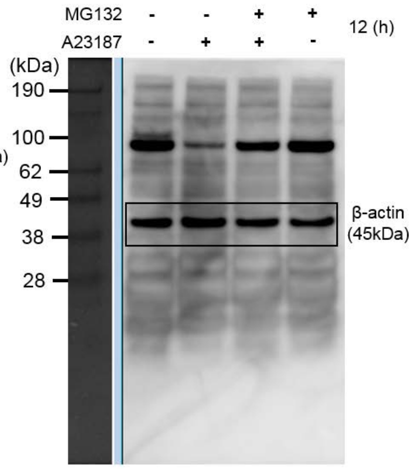

Figure 2B.

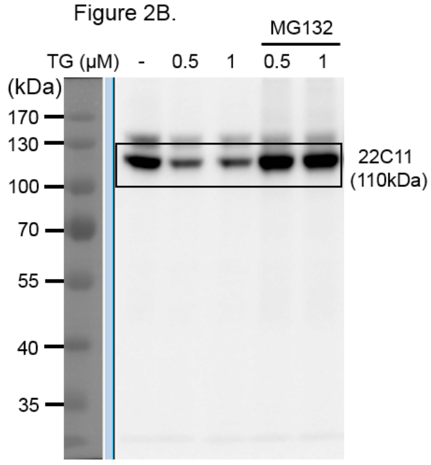

Figure 2B.

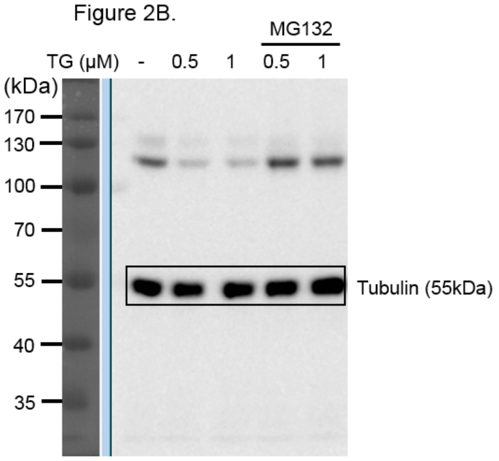

Figure 2C.

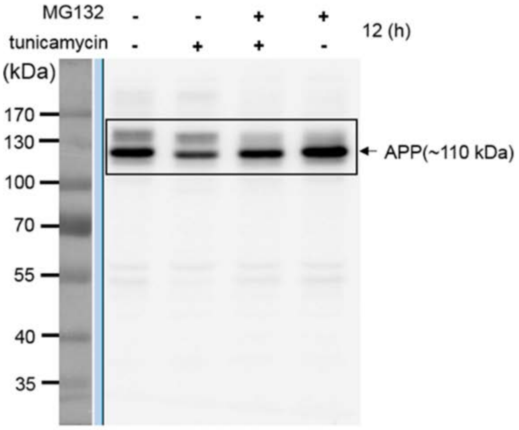

Figure 2C.

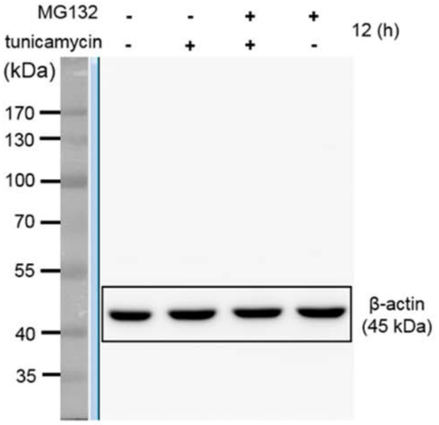

Figure 2D.

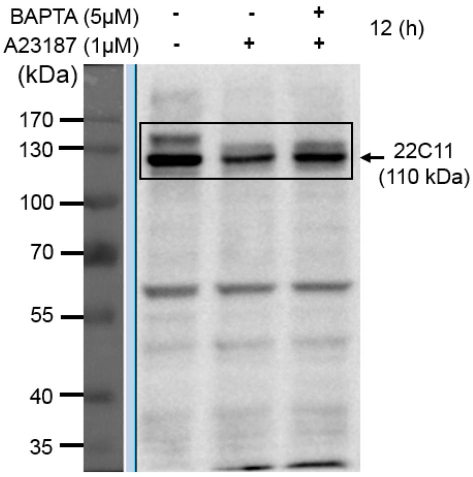

Figure 2D.

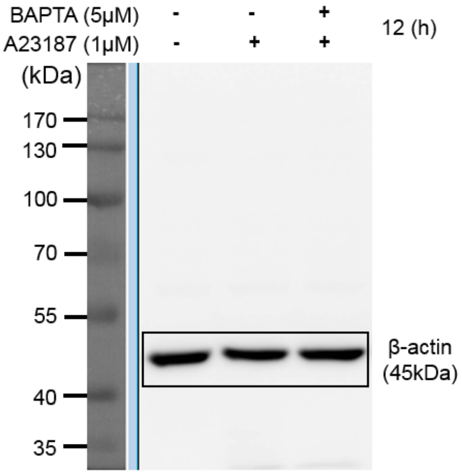

Figure 3A.

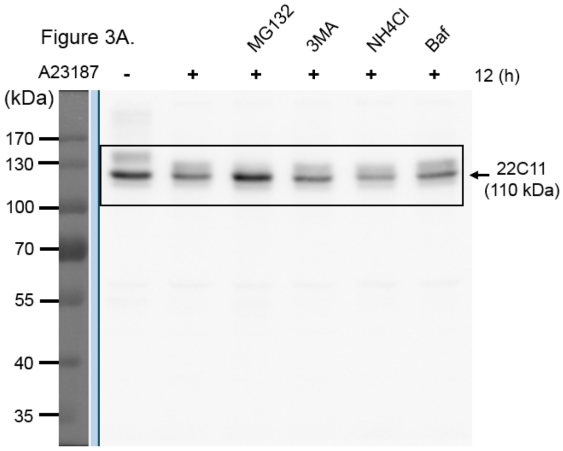

8% Glycine gel

Figure 3A.

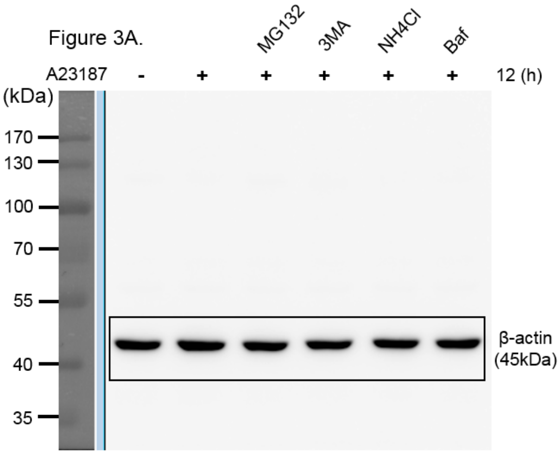

Figure 4B.

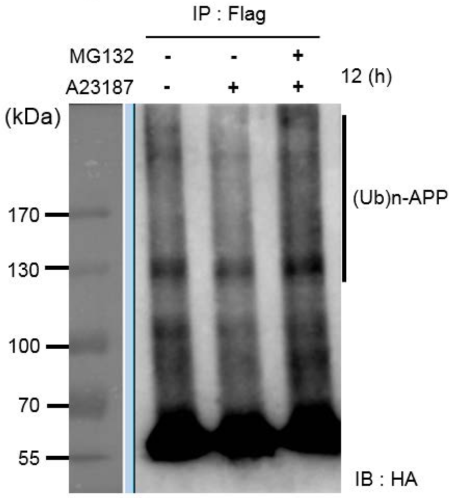

6% Glycine gel

Figure 5A.

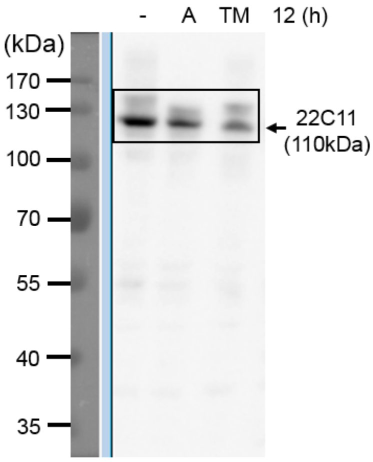

8% Glycine gel

Figure 5A.

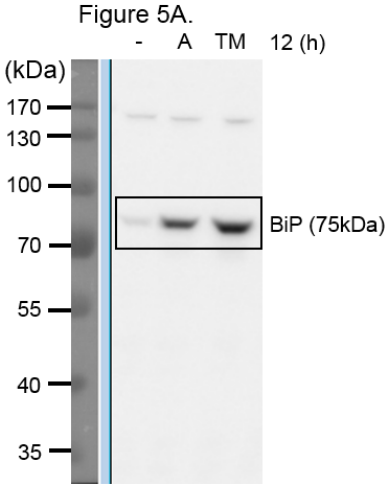

Figure 5A.

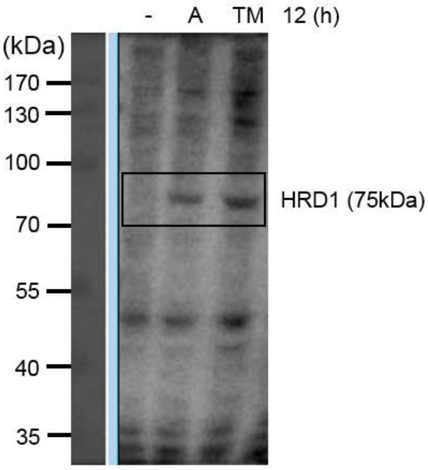

Figure 5A.

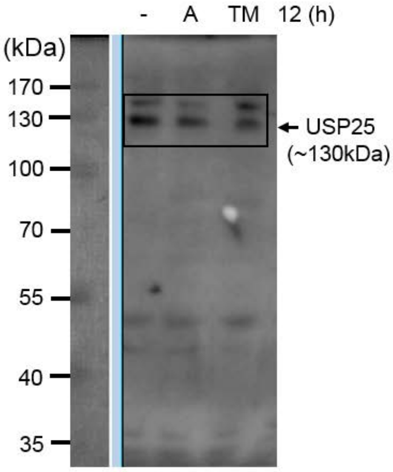

Figure 5A.

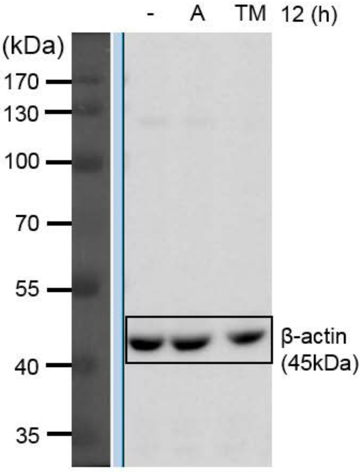

Figure 5B.

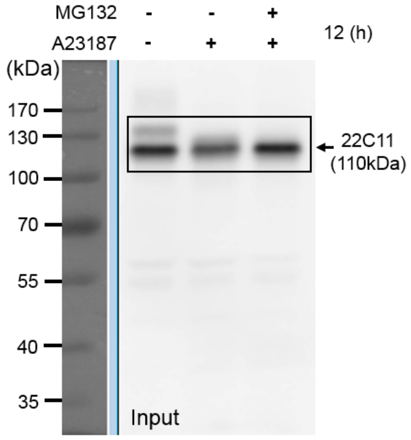

8% Glycine gel

Figure 5B.

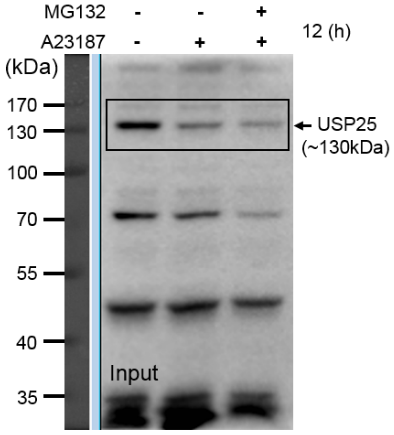

Figure 5B.

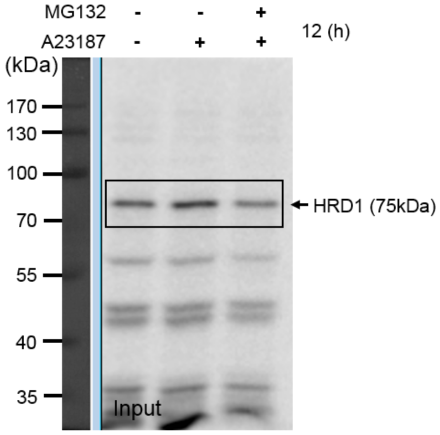

Figure 5B.

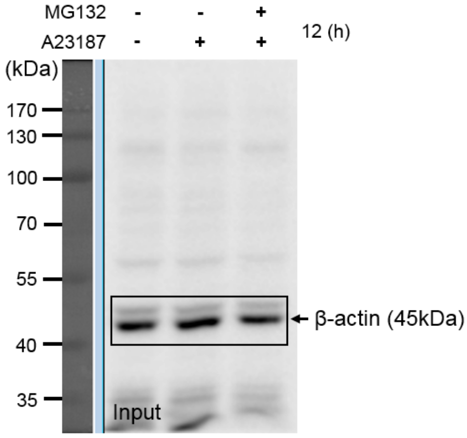

Figure 5B.

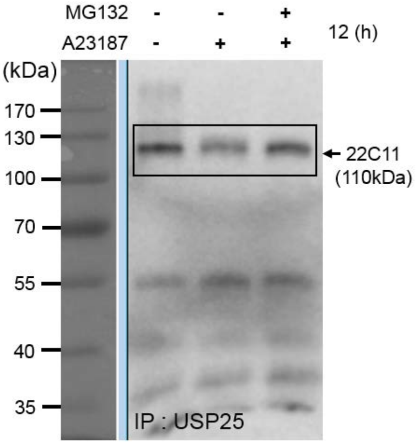

8% Glycine gel

Figure 5C.

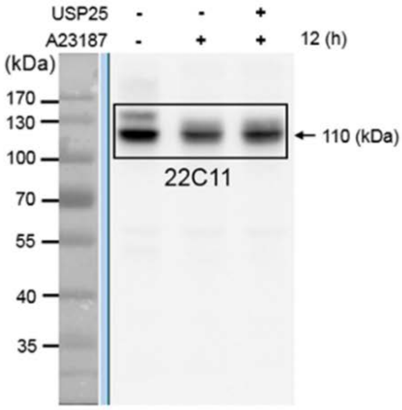

8% Glycine gel

Figure 5C.

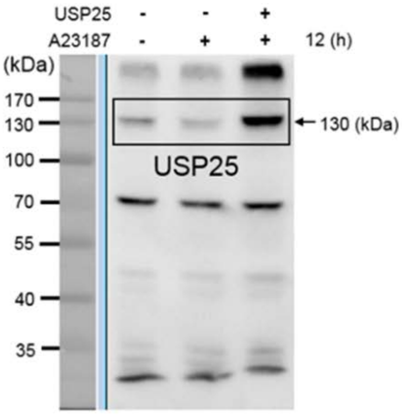

Figure 5C.

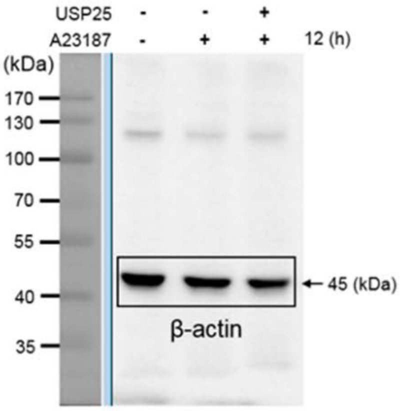

Figure S2.

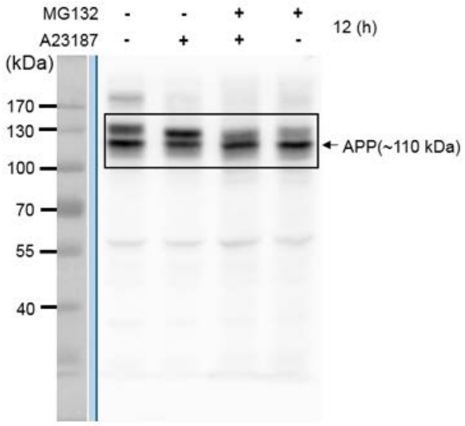

8% Glycine gel

Figure S2.

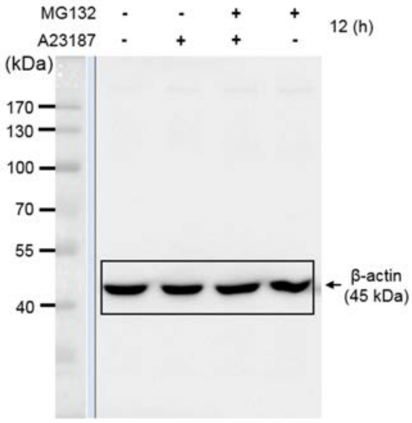

Figure S3.

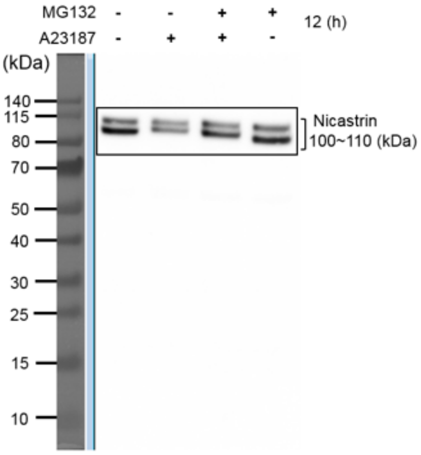

NuPAGE (4-12%)

Figure S3.

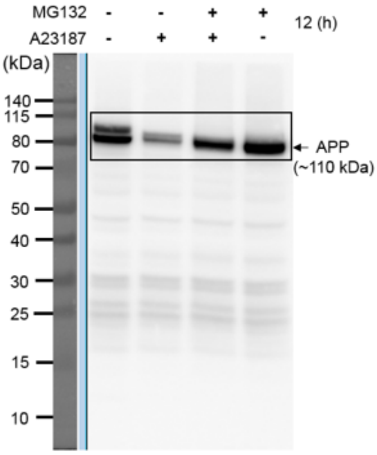

Figure S3.

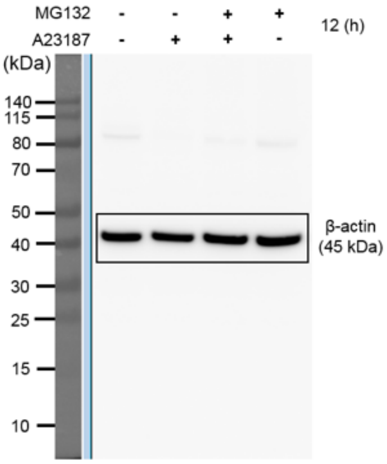

Supplement: Supplementary Information [file srep08805-s1.pdf]
